# Human rights-based mental healthcare: new developments, introducing a themed series of papers

**DOI:** 10.1192/bjo.2026.12022

**Published:** 2026-07-08

**Authors:** Helen Herrman, Paul S. Appelbaum, Silvana Galderisi, Norman Sartorius, John Allan, Martha Savage, Maria Rodrigues, Neeraj Gill, Guadalupe Morales Cano

**Affiliations:** Centre for Mental Health, The University of Melbourne, Australia; Department of Psychiatry, Columbia University, New York, USA; Department of Psychiatry, University of Campania Luigi Vanvitelli, Italy; Association for the Improvement of Mental Health Programmes (AMH), Geneva, Switzerland; Mayne Academy of Psychiatry, Faculty of Medicine and Biomedical Sciences, The University of Queensland, Australia; School of Geography, Environment and Earth Sciences, Victoria University of Wellington, New Zealand; School of Medicine, Griffith University, Australia; Mental Health Policy Unit, Health Research Institute, Faculty of Health, University of Canberra, Australia; International Bipolar Foundation, Madrid, Spain; Kindred Collaborative, Melbourne, Australia

**Keywords:** Human rights, mental health, health professionals, training, care

## Abstract

Mental healthcare respecting human rights is a worldwide need, yet research into practices that support such rights is limited. The United Nations Convention on the Rights of Persons with Disabilities, 2006 and the United Nations Resolution on mental health and psychosocial support, 2023 each heighten the urgency and the legal, as well as moral, social, political and other obligations to improve the quality of mental healthcare and respect human rights worldwide. It is useful to be specific about the actions to be taken, as done in recent programmes by the World Psychiatric Association and the World Health Organization. The work requires partnerships at all levels, from global to local, among healthcare professionals, people with lived experience and their families, communities and policy-makers. We present a themed series of papers developed in two parts: one related to principles of human rights-based mental healthcare; the other to assessment, policy and actions needed for tackling the implementation gap.

**Figure:**
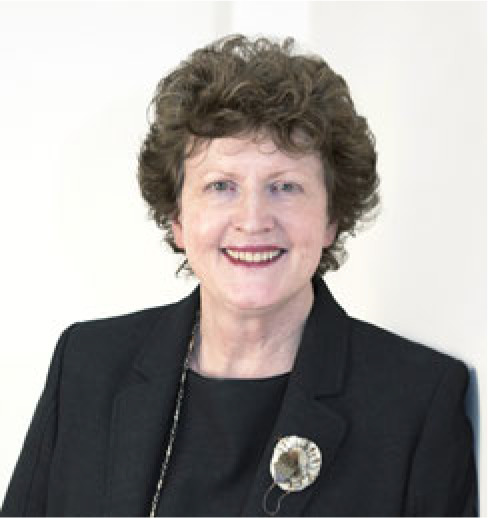

Mental healthcare respecting human rights is an urgent need for communities and the mental health professions worldwide. Nevertheless, research on practices that support human rights is limited. The adoption of the United Nations Convention on the Rights of Persons with Disabilities (CRPD), 2006 and the United Nations Resolution on mental health and psychosocial support, 2023 represent international obligations and commitments that heighten the urgency to improve mental healthcare across the world, and to respect and promote rights (^1^, pp. 5–16). We are pleased to respond accordingly with a themed series of papers from authors active in this work. The series is developed in two parts: one related to principles of human rights-based mental healthcare; the other to policy and actions needed for change. This reflects a dual challenge in contemporary mental healthcare: first, acknowledging the legal as well as moral, social, political and other obligations relevant to human rights in mental healthcare; and second, tackling the implementation gap, including the need for measurement and monitoring of change.

The submission of several of the papers is linked with two programmes developed by the World Psychiatric Association (WPA). The creation of an international initiative on implementing alternatives to coercion directly involves persons with lived experience of mental health conditions, their families and carers and mental health professionals, including psychiatrists.^2^ Aligned with this is the WPA programme to produce and adopt a universal code of ethics for use in the work of psychiatrists and the governance of mental healthcare worldwide.^3^ An international network of people recently engaged in this work are among those who have contributed papers. Although recognising that legal and practice traditions vary in different parts of the world (e.g. between the code-based systems influenced by the Francophone legal tradition and the common law systems influenced by Anglophone work and laws), we hope this series will support a worldwide commitment to change and encourage all involved in mental healthcare to become more aware of the resources available and the possibilities for change.

An editorial from Stavert and Szmukler draws on their own work and the expert scoping review prepared for this issue by McSherry and colleagues to comment on human rights promotion and the need for reform.^4,5^ Individual autonomy on an equal basis with others is a fundamental right for people with lived experience of mental healthcare and treatment. We believe this to be incontestable, and that autonomy is essential for protecting other human rights. Human rights cover an interconnected range of civil, political, economic, social and cultural rights. Given this, how best to protect these rights is a topic that requires urgent attention. If a person with mental disability is to live the best life they can, on their own terms, the full range of their human rights must be recognised because these underpin the enablement and necessary support required to achieve this. CRPD details those rights. Its model is that the degree to which a physical or mental impairment is a disability depends on the extent to which society makes reasonable accommodations to the impairment. A person’s diagnosis or related impairment must not justify inequalities in the enjoyment of those rights (^4^, p. 1).

Although many countries have ratified CRPD, the only binding human rights treaty focusing specifically on disability, and laws have been amended in a number of countries, its implementation is slow. There is a question of whether the rights to health and life may conflict with those to freedom and autonomy. A strong commitment to listening to the people who use services and their families and carers, and to providing a range of alternatives to non-consensual measures, is the first step. Supported decision-making, advance directives and a range of training, educational, practice and policy measures can be used to respect individuals’ will and preferences, rather than relying on coercive practices, thereby upholding all rights, including the right to health and the right to life. A partnership with health professionals that fully involves people with lived experience of mental health conditions and their families and carers is more likely than any group acting alone to generate support for reform from the political and policy community.^6^

An innovative study from Gronholm and colleagues refers to the challenge of how to prioritise the push for human rights.^7^ Protecting all human rights of people with mental health conditions is of prime importance globally. However, in regard to the practical implementation of rights-based care, the authors consider that it may be necessary to focus first on a few of them. The study used the Priorities of Human Rights and Mental Health approach in mental healthcare settings. The study sample included persons with lived experience of mental health conditions, as well as experts in the fields of human rights, mental health law, public health, psychiatry, medical ethics, disability support and advocacy. Three rights were consistently ranked as top priorities: (a) the right to freedom from torture, cruel inhuman treatment and punishment; (b) the right to health and access to services/treatment; and (c) the right to protection and safety in emergency situations. The study found overall a mix of the so-called negative rights (protecting persons’ civil and political rights from abridgment) and positive rights (providing persons with something, e.g. economic, social and cultural rights) as top priorities, affirming the importance of both. Supporting the positive rights is a crucial part of the needed changes. Improving and regular monitoring of the living conditions of people in residential or in-patient facilities are among urgent reforms noted in CRPD, as is a central voice for people with lived experience in research and implementation of decisions about the priority of human rights.

Stakeholders worldwide agree that coercion is overused in mental healthcare. Its widespread application and documented harms are also well understood.^4,8^ However, professional groups including psychiatrists remain divided about the path to reform. Gill and colleagues provide a joint perspective from the World Health Organization (WHO) work on the Quality Rights initiative and the WPA initiative on implementing alternatives to coercion that is groundbreaking in this regard.^9^ They discuss the organisations’ mutual aim to support countries in observing human rights and improving the quality of care, as well as the differences between their stated goals related to coercion in mental healthcare. The WHO’s approach to elimination of coercion aims to avoid the situation in which ‘exceptions’ become the rule, and to ensure that coercive practices are seen as something negative and to be avoided. The WPA position calls for practical system and service changes that support alternatives to coercion and increase observance of human rights, without specific mention of the elimination of coercion. However between them they conclude that focusing on an agreed need to find practical solutions has the power to build consensus and unify key actors. Both organisations seek to support persons with lived experience, families and carers, mental health professionals and policy-makers coming together to work towards the common goals of improving quality, promoting human rights and addressing coercion in mental health services. WHO has released new documents to support this work,^9^ and WPA continues advocacy with member societies to encourage them to support each other in amending and implementing reform.^1^

The second set of papers has a growing list of titles from different countries and regions. Savage and colleagues describe the most comprehensive attempt to date to compare rates of coercive practices in different countries.^10^ They devised methods of making relevant comparisons despite the widely varying monitoring strategies in use. They found that the rates and durations of seclusion and restraint differed by factors of more than 100 among countries, concluding that it is unlikely that such marked differences can be attributed solely to reporting differences. They recommend a common set of international measures to enable finer comparisons within and between countries, and monitoring of trends to determine whether alternatives to restraint are successful.

Fellinger and colleagues reach conclusions with similar relevance.^11^ They examine the association between COVID-19 lockdowns and involuntary psychiatric admissions in Austria. Their description of fewer but longer involuntary psychiatric admissions during the weeks of lockdown strengthen previous observations on the influence of external factors on the use of coercion. Even when services seek to avoid coercive measures such as involuntary psychiatric admission, numerous factors are known to influence their use. These findings highlight the need to clarify the causality and consequences of using coercive measures and the salience of alternatives to coercion.

An exemplary study on the quality of care and respect of human rights in mental health services in four West African countries describes an evaluation using the WHO QualityRights Toolkit.^12^ Despite reports that patients in West African psychiatric facilities are exposed to poor quality of care and human rights violations, evidence is lacking on the extent and profile of specific deficits in the services. The study concludes that inadequate appreciation of patients’ rights, lack of basic approaches to protect them and the failure to promote rights-based services in these facilities are major problems to be addressed. Although it recognises the resource constraints and need for more human and financial resources, the study also identifies critical changes required at the facility level, some of which can be implemented without major change in material resourcing. This is a theme picked up in several of the included papers. These cover emergency department and community settings as well as hospital wards, illuminating concerns about contraventions of human rights and inequity in all of these.

Three reports from high-income countries strengthen the understanding of vital components of programmes designed to improve or reduce restrictive practices.^13–15^ These include staff training in behaviour management and de-escalation, psychological support to patients and staff, effective staff–patient communication and the availability of alternatives. Above all is the need to integrate the experience of patients into staff training and coercion-reduction programmes. These studies note the major limitations of emergency departments in regard to crisis care for people with complex emotional needs, and the lack of research exploring alternatives. They comment on the value of a high-quality therapeutic relationship and of collaborative and optimistic staff, while noting that staff feel poorly supported in responding to needs. Research is needed into the experiences of a range of care options and how to improve these. This reminds us that respect for the human rights of people with mental health conditions is pivotal. Medical and psychiatric practices must adhere to respect for human rights, mental health facilities need to be monitored for use of coercive practices, and the satisfaction of patients evaluated; and, at the same time, mental health professionals need adequate training and support to provide rights-based care. Although recent publications, including WHO guidance on community mental health services (see WHO website), contain multiple examples of implementing rights-based and person-centred approaches, and a number of tools, including Safewards, Six Core Strategies, Open Doors and the WHO QualityRights toolkit, have been developed to assist in this work (^5^, and see the WPA website for an index of tools and resources), there is limited research and relatively few peer-reviewed publications in this critical field of reform.

Corderoy and colleagues’ study strengthens the evidence linking diagnostic, socioeconomic and cultural factors to involuntary treatment, and offers an important reminder for both policy-makers and practitioners: targeted interventions are needed to reduce involuntary admissions in disadvantaged groups.^16^ Several studies and reviews report on the use of compulsory treatment orders, including community treatment orders. These orders are applied in several countries to mandate treatment^17–21^ and hence are inherently coercive in nature even though applied in community settings. Studies of legislation, practice changes and variations in rates of use by region and diagnosis point to the urgent need for review of this practice.

Although the series focuses on the urgent need for reform here and now, Lepping and Poole remind us of the need to come to terms with the past, including shameful episodes in recent and more distant times.^22^ The process of analysing and accepting psychiatry’s past can help the profession get closer to its real self and on a path to a better future.^22^ Coming to terms with the past is a prerequisite for psychiatrists and other practitioners forming constructive partnerships with stakeholders – people with lived experience of mental ill health, their families, community groups and decision-makers – that are essential for improving mental healthcare.^2^ It is also necessary to be specific about the actions to be taken and to frame these as implementing alternatives to coercion. One way to advance is to study real-life experiences. Gill and Sartorius illustrate the value of working with others to meet the challenges of CRPD to mental healthcare, as well as the pressing need globally for research, evaluation and sharing experiences, as in the papers in this series.^1^ People in all countries, including disadvantaged and minority groups within countries, need rights-based care.

## Data Availability

Data availability is not applicable to this article because no new data were created or analysed in this study.
